# Accuracy and Reproducibility of Facial Measurements of Digital Photographs and Wrapped Cone Beam Computed Tomography (CBCT) Photographs

**DOI:** 10.3390/diagnostics11050757

**Published:** 2021-04-23

**Authors:** Maged Sultan Alhammadi, Abeer Abdulkareem Al-mashraqi, Rayid Hussain Alnami, Nawaf Mohammad Ashqar, Omar Hassan Alamir, Esam Halboub, Rodolfo Reda, Luca Testarelli, Shankargouda Patil

**Affiliations:** 1 Orthodontics and Dentofacial Orthopedics, Department of Preventive Dental Sciences, College of Dentistry, Jazan University, Jazan 45142, Saudi Arabia; 2 Department of Maxillofacial Surgery and Diagnostic Sciences, College of Dentistry, Jazan University, Jazan 45142, Saudi Arabia; abeerradiology@gmail.com (A.A.A.-m.); mhelboub@gmail.com (E.H.); 3 Internship Program, College of Dentistry, Jazan University, Jazan 45142, Saudi Arabia; alnami1122@hotmail.com (R.H.A.); nawaf6679@gmail.com (N.M.A.); o1414a@hotmail.com (O.H.A.); 4 Department of Oral Medicine, Oral Pathology and Oral Radiology, Faculty of Dentistry, Sana’a University, Sana’a 1247, Yemen; 5 Department of Oral and Maxillofacial Sciences, Sapienza University of Rome, 00185 Rome, Italy; rodolforeda17@gmail.com (R.R.); luca.testarelli@uniroma1.it (L.T.); 6 Division of Oral Pathology, Department of Maxillofacial Surgery and Diagnostic Sciences, College of Dentistry, Jazan University, Jazan 45142, Saudi Arabia; dr.ravipatil@gmail.com

**Keywords:** cone beam computed tomography, facial photographs, standardized photograph, wrapped photographs

## Abstract

The study sought to assess whether the soft tissue facial profile measurements of direct Cone Beam Computed Tomography (CBCT) and wrapped CBCT images of non-standardized facial photographs are accurate compared to the standardized digital photographs. In this cross-sectional study, 60 patients with an age range of 18–30 years, who were indicated for CBCT, were enrolled. Two facial photographs were taken per patient: standardized and random (non-standardized). The non-standardized ones were wrapped with the CBCT images. The most used soft tissue facial profile landmarks/parameters (linear and angular) were measured on direct soft tissue three-dimensional (3D) images and on the photographs wrapped over the 3D-CBCT images, and then compared to the standardized photographs. The reliability analysis was performed using concordance correlation coefficients (CCC) and depicted graphically using Bland–Altman plots. Most of the linear and angular measurements showed high reliability (0.91 to 0.998). Nevertheless, four soft tissue measurements were unreliable; namely, posterior gonial angle (0.085 and 0.11 for wrapped and direct CBCT soft tissue, respectively), mandibular plane angle (0.006 and 0.0016 for wrapped and direct CBCT soft tissue, respectively), posterior facial height (0.63 and 0.62 for wrapped and direct CBCT soft tissue, respectively) and total soft tissue facial convexity (0.52 for both wrapped and direct CBCT soft tissue, respectively). The soft tissue facial profile measurements from either the direct 3D-CBCT images or the wrapped CBCT images of non-standardized frontal photographs were accurate, and can be used to analyze most of the soft tissue facial profile measurements.

## 1. Introduction

The first photograph used in the medical field dated back to 1845 [1]. Thereafter, several advances in this practice were developed. Nowadays, dental photography is a routine clinical practice, and is a fundamental source of information with diverse uses. For example, soft tissue facial photographs are essential records for analyzing the maxillofacial region in many disciplines including orthodontics, orthognathic surgery, and facial plastic surgery, and for different purposes including diagnostic processing, treatment planning, and analysis of outcomes results [2,3]. There are two types of photographs in this regard: non-standardized and standardized facial photographs. The latter one is a more reliable tool for proper diagnosis, treatment planning, and treatment decision [4]. The diagnostic imaging technology has witnessed a giant revolution in the last three decades. With the introduction of 3D imaging, the subjects can be visualized in all planes rather than using a two-dimensional evaluation [5]. CBCT has changed the way dentistry is practiced since 1988. It is a valuable modality that precisely evaluates of skeletal components in the craniofacial region with a 1:1 image (no magnification). However, it is of limited value in the assessment of soft tissue facial characteristics [6,7].

Many studies [8,9] have evaluated the potential correlation between craniofacial measurements obtained from the gold standard cephalometric radiographs and analogous measurements from standardized facial profile photographs. They found the standardized photographic method to be repeatable and reproducible. Further, they considered it to be a feasible and practical non-invasive alternative diagnostic method so long as the standardized protocol is followed. Another study concluded that the soft tissue analysis on photographs is a reliable method to evaluate the soft tissue profile compared to the analyses performed on cephalograms [10].

Another recent method for recording the soft tissue profile is “stereophotogrammetry”. Although this method is almost accurate in representing the facial soft tissue compared to the direct anthropometric and the 2D standardized photogrammetric measurements, it needs expensive equipment, is time consuming, and takes considerable clinical workspace [11,12,13]. For their part, the standardized facial photographs require special types of equipment, precise stepwise technique, and more time than that required for 3D stereophotogrammetry [11,12,13].

There is a need to replace the expensive, and time- and space-consuming methods—3D stereophotogrammetry and standardized facial photographs—with a simple alternative utilizing CBCT, when indicated for diagnosis and treatment-planning purposes, to provide a 3D scan upon which both the hard and soft tissue can be measured to get precise and comprehensive diagnostic data. In this context, there is limited evidence that CBCT scans can act as a platform for evaluating the accuracy of morphed non-standardized random facial photographs. Therefore, this study sought to assess whether the soft tissue facial profile measurements of direct CBCT soft tissue and wrapped CBCT images of non-standardized facial photographs are accurate and reliable compared to the standardized digital photographs.

## 2. Materials and Methods

This cross-sectional study was approved by the Internal Review Board of Jazan University (15 November 2018) and the registration no. is (18191-2018). All patients signed informed consents ahead of registration in the institute database where they agreed that any patient data could be used for research purposes, including the use of human images.

The sample size was calculated with an alpha value of 0.05 and a power of 80% based on the study conducted by Mehta et al. [8]. They reported means and standard deviations of ANB° of 5.89 ± 1.45 and 6.38 ± 1.53 in cephalometric and standard photographic methods, respectively. The calculation showed a minimum sample of 59 subjects needed in this study. In total, 60 patients were included. All patients routinely referred for CBCT scans were checked for the following inclusion criteria: (1) male patient, (2) age between 18 and 30 years, and (3) stable occlusion (proper posterior intercuspation). Patients who had any of the following were excluded: (1) history of dentofacial trauma, (2) congenital syndromes causing facial deformity, (3) facial asymmetry, and/or (4) previous orthodontic or surgical treatment.

The CBCT scan was done using a standardized next-generation i-CAT CBCT machine (Imaging Sciences International, Hatfield, PA, USA). The image acquisition parameters were as follows: large field of view (17 cm) at 120 kV, 18.54 mAs, and 8.9-s exposure time. During scanning, the Frankfort horizontal plane was made parallel to the floor with a crossing laser guide. Patients were instructed not to swallow during the scan. Digital Imaging and Communications in Medicine (DICOM) files of the CBCT images were obtained.

**Figure 1:**
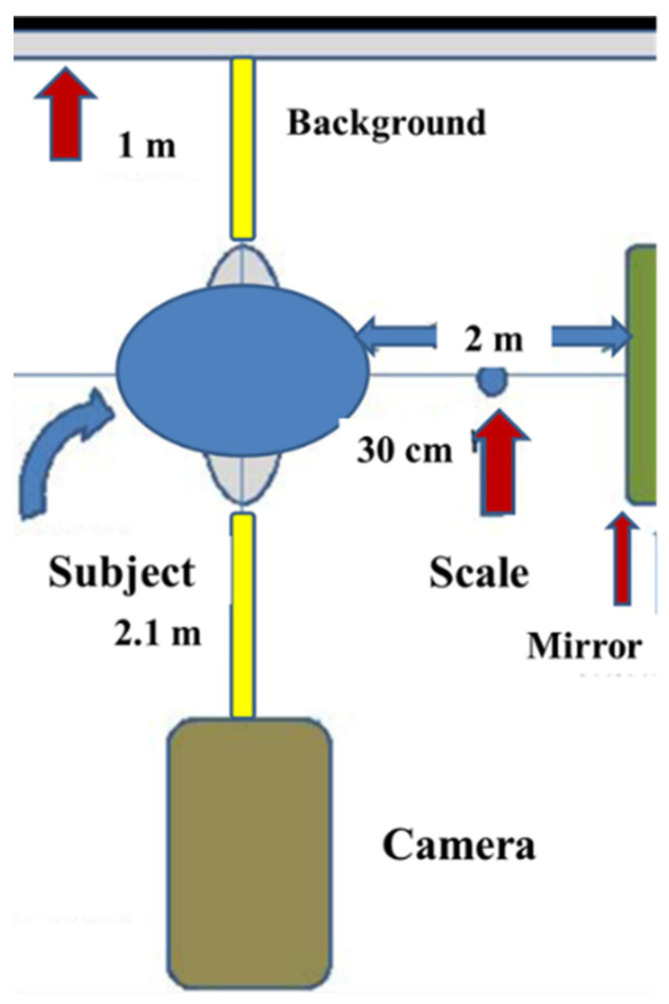
The parameters of the protocol used in the 2D standardized facial photography.

**Figure 2:**
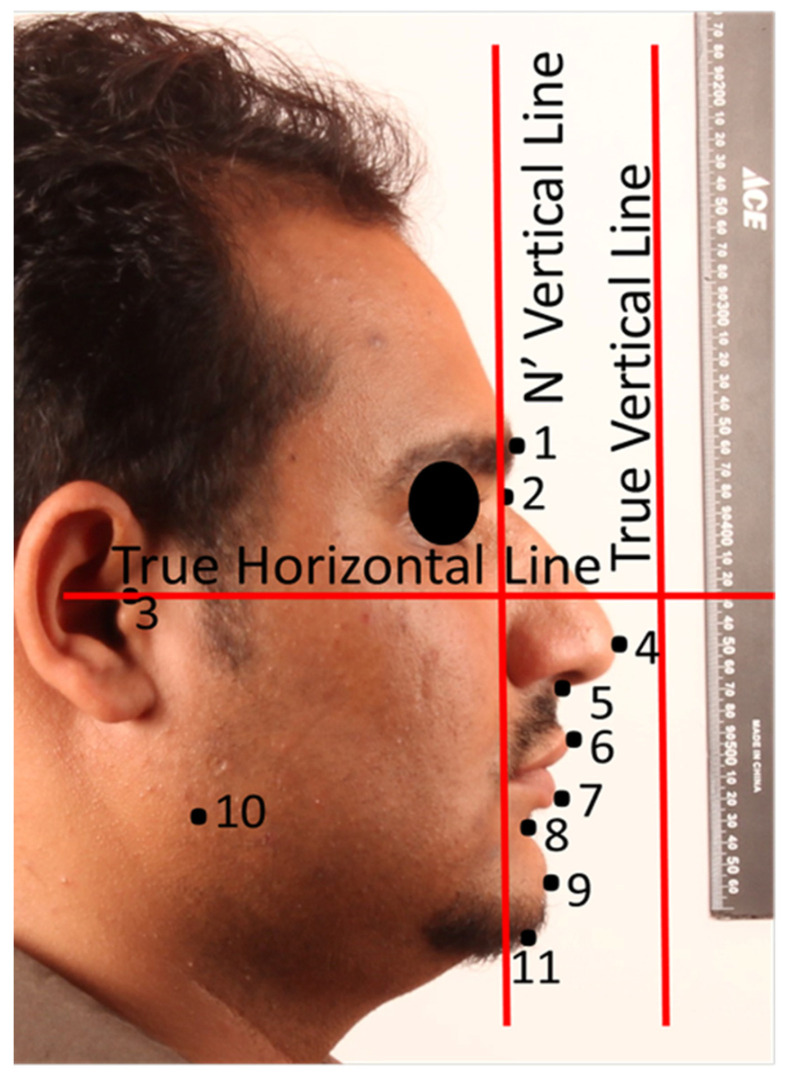
The facial profile soft tissue landmarks and main reference planes used in the study.

The participants were photographed using a precise protocol (Figure 1) [9]. For that purpose, a professional digital camera (EOS Digital Rebel XT, Canon, Tokyo, Japan) mounted with the same lens (EF 100 mm f/2.8 USM Macro Lens, Canon, Tokyo, Japan) and flash (Macro Ring Lite MR-14EX flash, Canon, Tokyo, Japan) was used in the manual position for all photographic records. A 100-mm macro lens was selected to avoid facial deformations and maintain natural proportions. The right profile photographs were standardized by using a reproducible set-up of a tripod, camera, and chair. Photographs were taken in the natural head position (NHP), with maximum intercuspation and the lips at rest. Glasses and any other obstacles, if necessary, were removed to obtain a clear image of craniofacial area for proper identification of points. A distance of 2.1 m was left between subject and the camera lens and 1 m was left between the subject and the background. The magnification of the image was calibrated using ruler on the anterior and upper side of the subjects. An adjustable mirror holder was used to hold a 35 × 70 cm mirror to allow for proper patient positioning at different heights. Another random set of facial frontal photographs (not standardized) were taken at different distances. The landmarks and reference lines used are presented in (Figure 2).

**Figure 3:**
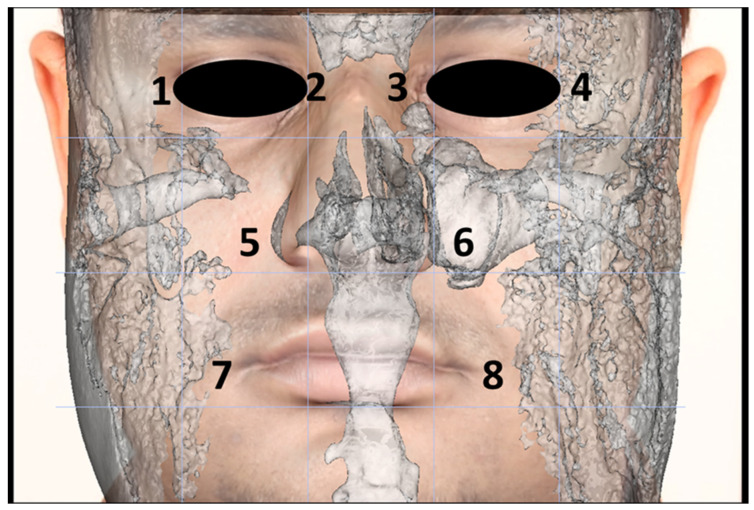
The eight CBCT wrapping points: (**1**) right lateral canthus, (**2**) right medial canthus, (**3**) left medial canthus, (**4**) left lateral canthus, (**5**) right nasolabial creases, (**6**) left nasolabial creases, (**7**) right oral commissure, and (**8**) left oral commissure.

The frontal non-standardized photograph was wrapped over the CBCT images using Anatomage 5.02 (Anatomage) using the “create face photo wrapping” icon (Supplementary file). Briefly, the soft tissue and bone windows were adjusted regarding the brightness property to show the full soft tissue 3D image. The threshold value was adjusted between 20 and 80 to minimize soft tissue artifacts. Thereafter, the frontal non-standardized photograph was inserted, and the frontal view of the 3D image was adjusted to be transparent, allowing for proper wrapping. The following eight points were made coincident for both the frontal non-standardized arbitrary photograph and the 3D CBCT images: the right and left lateral and medial canthi of the eyes, the right and left nasolabial creases, and the right and left oral commissures (Figure 3).

**Figure 4:**
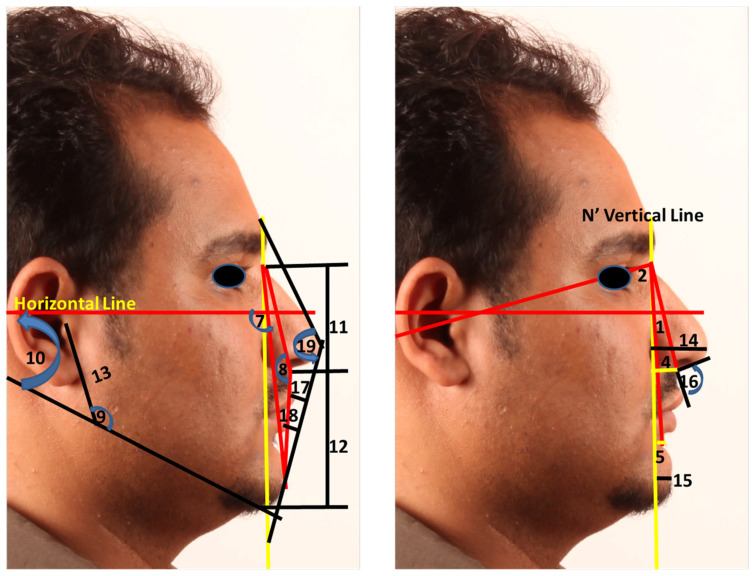
Showing the measurement used in the study as defined in Table 1: (**1**) Trg N’ .Sn. (°), (**2**) Trg N’ .B’ (°), (**3**) the difference between 1 and 2, (**4**) N’ Vertical Sn (mm), (**5**) N’ Vertical B’ (mm), (**6**) the difference between 5 and 6, (**7**) N’ Pog’/TH (°), (**8**) N’.Sn.Pog’ (°), (**9**) Trg. Go’.Me’ (°), (**10**) TH-MA (°), (**11**) AFH (N’-Me’) (mm), (**12**) LAFH (Sn-Me) (mm), (**13**) PFH (Trg-Go) (mm), (**14**) Pn-N’ Vertical (mm), (**15**) Pog’-N Vert (mm), (**16**) NLA (°), (**17**) Ls/E line (mm), (**18**) Li/E line (mm), and (**19**) Gb.Pn.Pog’ (°).

**Figure 5:**
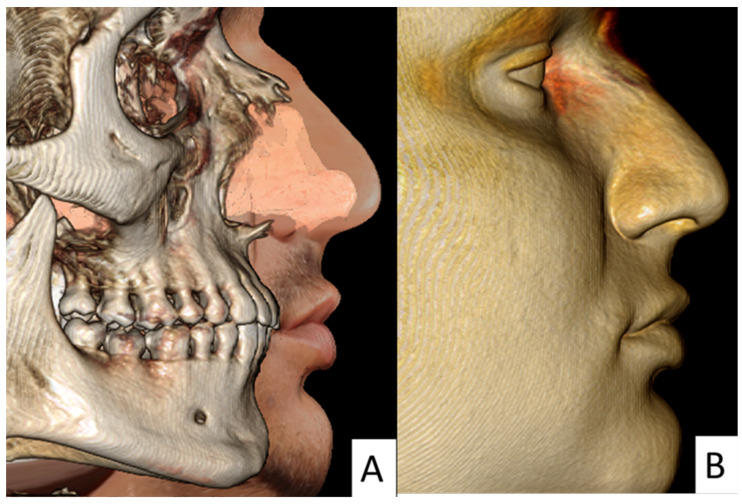
(**A**) Wrapped lateral profile view showed the profile view of the wrapped non-standardized photograph following the fixation of the selected soft tissue registration points. (**B**) Lateral direct CBCT soft tissue view.

The standardized facial profile measurements (Figure 4) were compared with non-standardized wrapped CBCT photographs and direct CBCT soft tissue rendering volumes (Figure 5). The facial soft tissue measurements used in this study are presented in Table 1.

The data were input, handled, and analyzed using Statistical Package for Social Sciences (SPSS) program version 25 (IBM Corp, North Castle, NY, USA). The variables were presented as means along with their corresponding standard deviations. In order to assess the reliability of measurements obtained by the “wrapped photograph” and the “direct CBCT soft tissue” methods, individually, against the “standard photograph”, Lin’s concordance correlation coefficients (CCC) were calculated and presented. The CCC values of >0.99, 0.95 to 0.99, 0.90 to 0.95, and <0.9 indicate “almost perfect”, “substantial”, “moderate” and “poor” reliability, respectively. In total, 10 CBCTs were selected randomly and measured independently by two examiners (A.A.A and M.S.A) on two occasions at 2-week intervals to assure the reliability of readings using the intraclass correlation coefficient (ICC).

The Bland–Altman plot (Bland and Altman, 1999)—a graphical method that compares two measurements methods where the differences (or alternatively the ratios) between the two methods are plotted against their averages—was conducted on two variables as examples for the purpose of visual approximation.

## 3. Results

Intra- and inter-examiner reliability was high ranging between 0.855 and 0.990. Descriptive statistics (means and standard deviations (SD)) of the different soft tissue variables measured by the standard photograph, wrapped photograph, and direct CBCT soft tissue are presented in Table 2.

The concordance correlation coefficients (CCC) showed that most of the soft tissue linear and angular measurements of maxillary and mandibular anteroposterior and vertical parameters were of high reliability (CCC ranged between 0.91 and 0.998), except for four soft tissue measurements which were unreliable: the posterior gonial angle (0.085 and 0.11 for wrapped and direct CBCT soft tissue, respectively), the mandibular plane angle (0.006 and 0.0016 for wrapped and direct CBCT soft tissue, respectively), the posterior facial height (0.63 and 0.62 for wrapped and direct CBCT soft tissue, respectively), and the total soft tissue facial convexity (0.52 for both the wrapped and direct CBCT soft tissue, Table 3).

**Figure 6:**
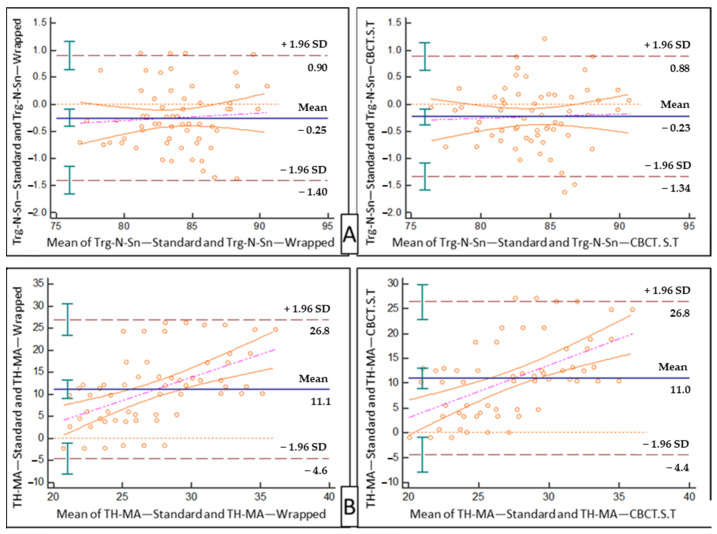
Two samples of Bland–Altman results. (**A**) The result of the two studied methods against the standard method for Trg N’ .Sn. (°). (**B**) The results of the two studied methods against the standard method for TH-MA (°).

Bland–Altman plots for the “wrapped photograph” and “direct CBCT soft tissue” methods, individually, against the “standard photograph” were drawn for Trg N’. Sn. (°) (Figure 6A), and for TH-MA (°) (Figure 6B).

## 4. Discussion

Obtaining a precise 3D facial model is of utmost importance to orthodontics, orthognathic, and plastic surgery. It does not only help the operator in proper real-time treatment decisions, but also helps in simulating treatment outcomes. On the patient’s side, this reduces the negative psychological influences of the selected treatment plan and shows the different alternatives. The best method to achieve this goal is to have the hard and soft tissues in a single model.

The main idea of the current study is that a random frontal photograph, even by using a cell phone camera, can be used for proper analysis of the soft tissue profile by utilizing a CBCT soft tissue platform, and can then be compared with the accuracy and reliability of each of the two CBCT soft tissue profile methods against the 2D standard profile photographs. Compared to the latter, each of these two methods showed excellent inter- and intra-observer agreement in most of the soft tissue facial profile measurements.

Several evaluation methods of the soft tissue profile are used: direct anthropometry, standardized 2D profile photography, 3D profile photogrammetry, standard lateral cephalometry, 3D stereophotogrammetry, and direct CBCT soft tissue analysis. Direct anthropometry and lateral cephalometry are the gold standards against which any new photographing method must be weighed.

The standardized 2D profile photographic method has been claimed as a reliable tool, De Carvalho et al. [9], compared this method against the gold standard cephalometric soft tissue profile method and reported a satisfactory reliability, and most of the used measurements showed ICCs above 0.80.

Three-dimensional soft tissue facial photographs, either by stereophotogrammetry or a 3D camera, can be wrapped over CBCT, with limited evidence about their accuracy. Almulla et al. [14] used a 3D camera to wrap photos in both 2D and 3D formats over the CBCT images in comparison with direct anthropometric measurements. In total, 23 out of 26 linear measurements were unreliable, suggesting that none of the three alternative methods could be a suitable substitute for the direct measurement method. The main disadvantages of the 3D camera are the high cost, possible image distortion by movement, time-consuming process, and lack of sufficient evidence in hand about their accuracy in clinical orthodontic practice [15,16].

The wrapping of random 2D photographs, if accurate, is a simpler and less expensive alternative. There is only one available study that used the same methodology, in which 2D photographs were wrapped on CBCT, but most of their measurements were on frontal soft tissue analysis, which is less important in the field of orthodontics [14].

The results of this study showed that the linear and angular anteroposterior maxillary and mandibular soft tissue measurements were of high reliability, with a CCC that ranged between 0.91 and 0.99. These measurements were not evaluated by Almulla et al. [14], except for one anteroposterior measurement—nasal tip protrusion.

The vertical parameters also showed high reliability, except for the posterior gonial angle, posterior facial height, and mandibular plane angle. This contradicts the results reported by Almulla et al. [14]; they found significant differences between the only two vertical measurements used in their study, the lower and the upper facial height. They did not explain the reason behind this inconsistency between both methods.

In our study, the unreliable vertical measurements mentioned were shared with a common landmark (Gn), which is difficult to identify in both the standard and the studied methods.

The reliability was also high for the soft tissue facial angle, nasal and chin anteroposterior position, upper and lower lip anteroposterior position, and nasolabial angle. These measurements were not evaluated by Almulla et al. [14], except for as mentioned earlier, the nasal tip protrusion, which they found unreliable. They used Sn-Prn as a reference. This indicates the nasal protrusion relative to the upper lip base, and not to the forehead, which is more commonly used in orthodontic practice and plastic surgery. However, the total soft tissue facial convexity was unreliable in our study, mostly due to a failure of allocation of the glabella points in some cases, especially those with long faces, as they become out of the field of view of the CBCT machine.

The major limitation of this study was the field of view of the CBCT machine. It was not enough to accurately locate the glabella region. Another limitation was that all the included subjects were males. Therefore, it is recommended to conduct more studies using larger field of view and including samples of both genders.

## 5. Conclusions

The use of wrapped CBCT images from non-standardized random frontal photographs is reliable and can be used for the purpose of analyzing the soft tissue facial profile measurements.

## Figures and Tables

**Table 1:** Soft tissue measurements used in the study.

Measurement	Abbreviation	Definition
Angular maxillary soft tissue position	Trg N’ .Sn. (°)	Angle between tragus (Trg), soft tissue nasion (N’), and the midpoint of the angle at the columella base (Sn).
Angular mandibular soft tissue position	Trg N’ .B’ (°)	Angle between tragus (Trg), soft tissue nasion (N’), and the deepest concavity between the vermilion border and the chin (B’).
Maxillomandibular angular discrepancy	Sn N’ .B’ (°)	Angle between Sn, N’ and B’.
Linear maxillary soft tissue position	N’ Vertical Sn (mm)	The linear distance between perpendicular line from N’ to the true horizontal line (N’ Vertical) and the Sn point.
Linear mandibular soft tissue position	N’ Vertical B’ (mm)	The linear distance between perpendicular line from N’ to the true horizontal line and the B’ point.
Maxillomandibular linear discrepancy	Sn-B perp (mm)	The linear differences between N’ Vertical Sn and N’ Vertical B’.
Facial soft tissue angle	N’ Pog’/TH (°)	Angle between N’ and the most anterior midpoint of the soft tissue of the chin with the true horizontal line.
Facial soft tissue convexity	N’.Sn.Pog’ (°)	Angle between N’, Sn, and Pog’.
Posterior gonial angle	Trg. Go’.Me’ (°)	Angle between tragus (Trg), soft tissue gonion (Go’), and the midpoint inferior point of the chin (Me’).
Mandibular plane angle	TH-MA (°)	Angle between the true horizontal plan and Go’-Me’ line.
Anterior facial height	AFH (N’-Me’) (mm)	Linear distance between N’ and soft tissue menton (Me’).
Lower anterior facial height	LAFH (Sn-Me) (mm)	Linear distance between Sn and soft tissue menton (Me’).
Posterior facial height	PFH (Trg-Go) (mm)	Linear distance between Trg and soft tissue gonion (Go’).
Nasal linear position	Pn-N’ Vertical (mm)	Linear distance between the most prominent point of the nose and N’ Vertical line.
Chin linear position	Pog’-N Vert (mm)	Linear distance between the most anterior midpoint of the soft tissue of the chin (Pog’) and N’ Vertical line.
Nasolabial angle	NLA (°)	Angle formed between the tangent to the base of the nose and the tangent to the upper lip.
Upper lip position	Ls/E line (mm)	Linear distance between the most prominent point in the vermilion border of the upper lip and the Esthetic line.
Lower lip position	Li/E line (mm)	Linear distance between the most prominent point in the vermilion border of the lower lip and the Esthetic line.
Total soft tissue facial convexity	Gb.Pn.Pog’ (°)	Angle between Glabella, Pn, and Pog’ points.

**Table 2:** Descriptive statistics (means and standard deviations (SD)) of the different variables measured by the standard photograph, wrapped photograph, and direct CBCT soft tissue.

Variable	Standard Photograph		Wrapped Photograph		Direct CBCT Soft Tissue	
	Mean	SD	Mean	SD	Mean	SD
Trg N .Sn. (^)	83.7	3.12	83.95	3.08	83.12	3.1
Trg N .B (^)	74.28	3.28	74.35	3.11	74.39	3.18
Sn N .B (^)	9.42	2.19	9.59	2.17	9.53	2.26
N Vertical Sn (mm)	18.02	6.11	18.89	6.26	17.53	6.3
N Vertical B (mm)	16.21	6.92	16.91	6.92	15.71	6.92
Sn-B perp (mm)	1.81	7.39	1.98	7.48	1.82	7.35
N Pg/TH (^)	93.53	5.13	94.63	5.13	92.83	5.13
N.Sn.Pog (^)	163.21	6.45	162.85	6.41	163.27	6.64
Tr. Go.Me (^)	144.11	8.43	130.97	5.98	131.66	6.21
TH-MA (^)	32.93	7.09	21.82	3.91	21.96	3.8
AFH (N-Me) (mm)	180.92	11.61	180.87	12.1	180.95	11.77
LAFH (Sn-Me) (mm)	108.05	9.86	108.08	10.3	108.16	10.23
PFH (Tr-Go) (mm)	77.39	12.15	90.39	12.15	90.69	12.15
Pn-N Vert (mm)	38.88	6.38	39.22	6.72	37.53	6.32
Pog-N Vert (mm)	13.53	7.27	14.53	7.3	13.08	7.25
NLA	104.62	9.82	105.15	9.81	104.8	9.77
Upper lip position (mm)	6.13	3.6	6.15	3.55	6.08	3.57
Lower lip position (mm)	2.9	2.43	3.05	2.42	3.05	2.43
Gb.Pn.Pog (^)	140.72	5.61	134.3	5.87	134.28	6.22

**Table 3:** The concordance correlation coefficients (CCC) reflecting the reliability of the measurements of the applied methods.

Variable	Standard Photograph vs. Wrapped Photograph		Standard Photograph vs. Direct CBCT Soft Tissue	
	Value	CI	Value	CI
Trg N .Sn. (^)	0.98	0.97–0.99	0.98	0.97–0.99
Trg N .B (^)	0.97	0.96–0.98	0.98	0.96–0.99
Sn N .B (^)	0.91	0.85–0.94	0.94	0.90–0.96
N Vertical Sn (mm)	0.98	0.97–0.99	0.99	0.985–0.995
N Vertical B (mm)	0.995	0.993–0.996	0.97	0.996–0.998
Sn-B perp (mm)	0.993	0.99–0.996	0.96	0.994–0.998
N Pg/TH (^)	0.977	0.967–0.984	0.997	0.987–0.994
N.Sn.Pog (^)	0.98	0.97–0.99	0.989	0.982–0.993
Tr. Go.Me (^)	0.085	−0.001–0.18 *	0.11	0.007–0.021 *
TH-MA (^)	0.006	−0.007–0.008 *	0.0016	−0.06–0.1 *
AFH (N-Me) (mm)	0.97	0.96–0.98	0.98	0.97–0.99
LAFH (Sn-Me) (mm)	0.96	0.93–0.98	0.96	0.93–0.97
PFH (Tr-Go) (mm)	0.63	0.54–0.71 *	0.62	0.53–0.7 *
Pn-N Vert (mm)	0.98	0.96–0.99	0.98	0.97–0.99
Pog-N Vert (mm)	0.99	0.986–0.993	0.996	0.994–0.998
NLA	0.98	0.96–0.99	0.99	0.98–0.993
Upper lip position (mm)	0.996	0.994–0.998	0.99	0.98–0.994
Lower lip position (mm)	0.994	0.99–0.996	0.99	0.98–0.993
Gb.Pn.Pog (^)	0.52	0.39–0.62 *	0.52	0.39–0.63 *

* Unreliable measurements.

## Supplementary Materials

The Supplementary Materials are available online at https://www.mdpi.com/article/10.3390/diagnostics11050757/s1.

## Abbreviations

2D	Two-dimensional
NHP	Natural Head Position
3D	Three-dimensional
SD	Standard Deviations
CBCT	Cone Beam Computed Tomography
SPSS	Statistical Package for Social Sciences
CCC	Concordance Correlation Coefficients
Sn Prn	Subnasale-pronasale
DICOM	Digital Imaging and Communications in Medicine
TH-MA	Mandibular plane angle
Gn	Gnathion
Trg N’. Sn	Angular maxillary soft tissue position
