# Supplementary material for: Accuracy and Reproducibility of Facial Measurements of Digital Photographs and Wrapped Cone Beam Computed Tomography (CBCT) Photographs
Source: Diagnostics (Basel). 2021 Apr 23;11(5):757. doi: 10.3390/diagnostics11050757 (PMC8147051; doi:10.3390/diagnostics11050757)

Steps of photo wrapping of non-standardized facial frontal photograph on CBCT 3D volume:

1) **Open create face photo wrapping window**

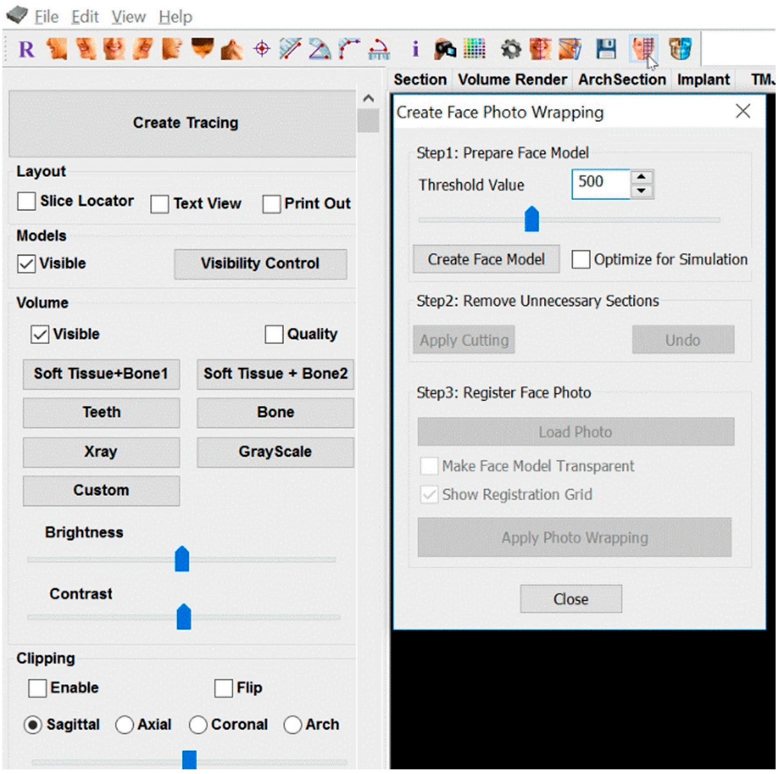

2) **Adjust the threshold value to be between 20 and 80 to minimize scattered soft tissue artifacts**

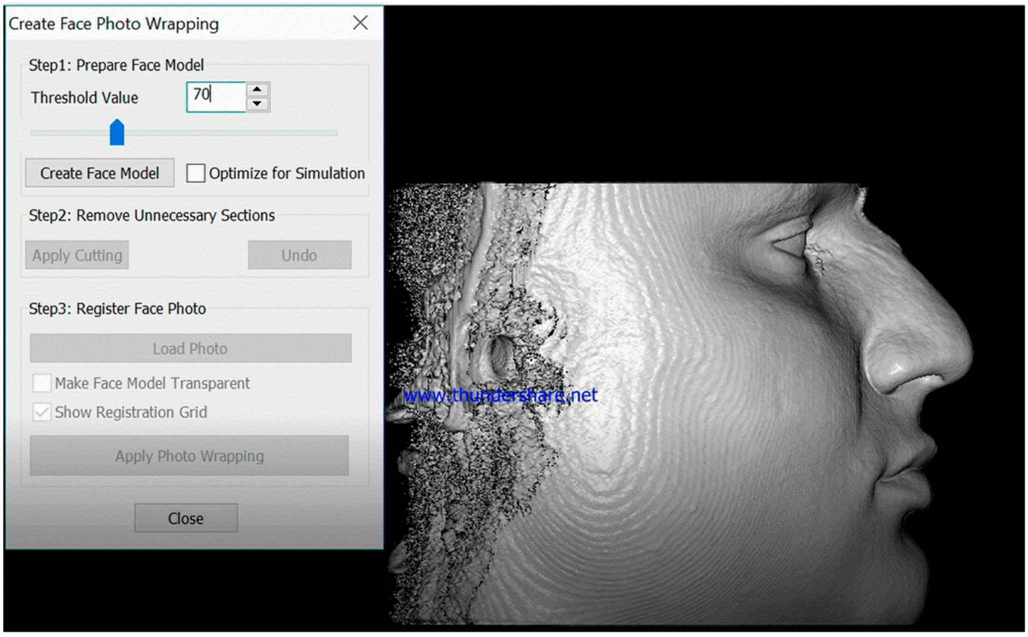

3) **Click on optimize for simulation then click on create face model**

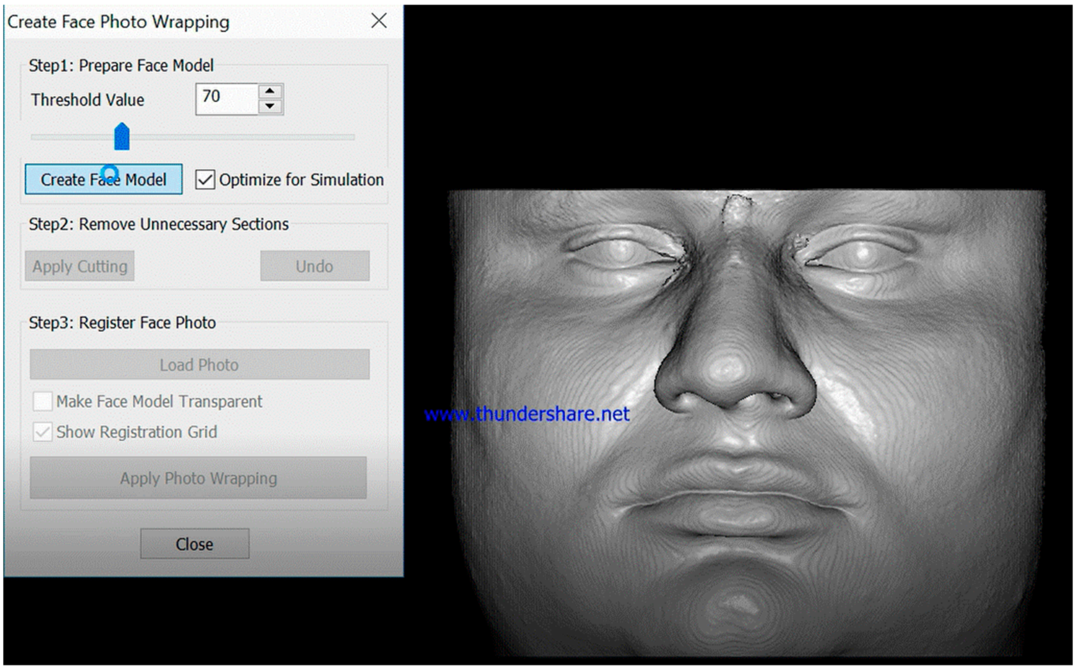

4) **Face model created**

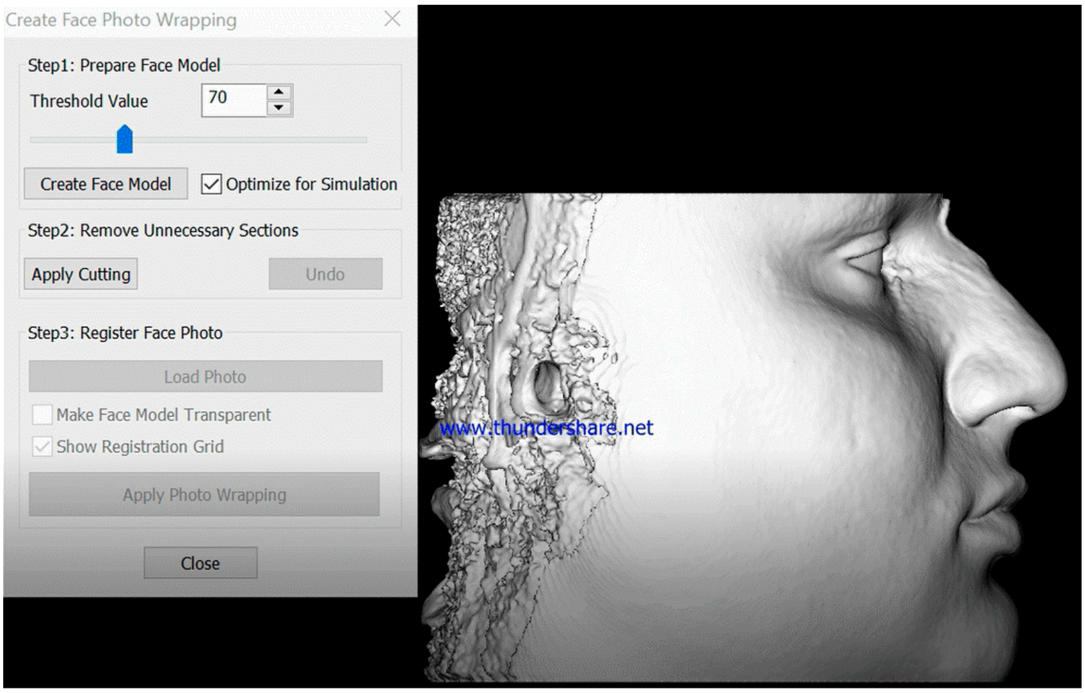

5) **Click on apply cutting to cut off 3D surfaces outside the region of interest**

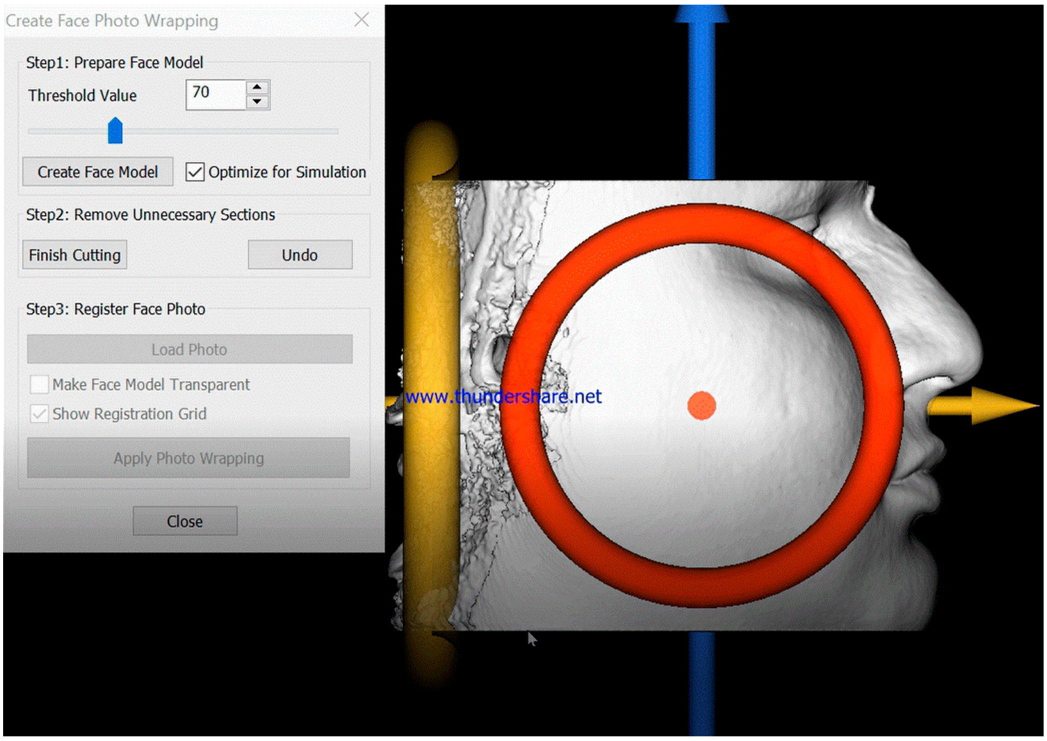

6) **Click on finish cutting once the region of interest is selected**

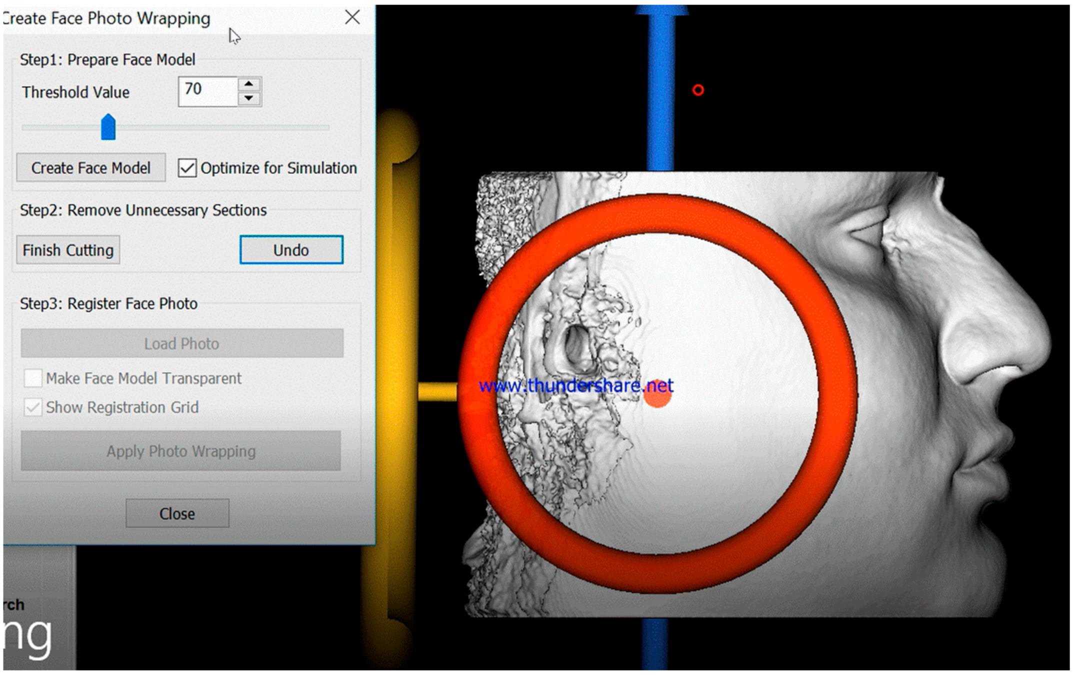

7) **Load the non-standardized facial frontal photograph and use the 3D CBCT soft tissue as a platform for adjustment**

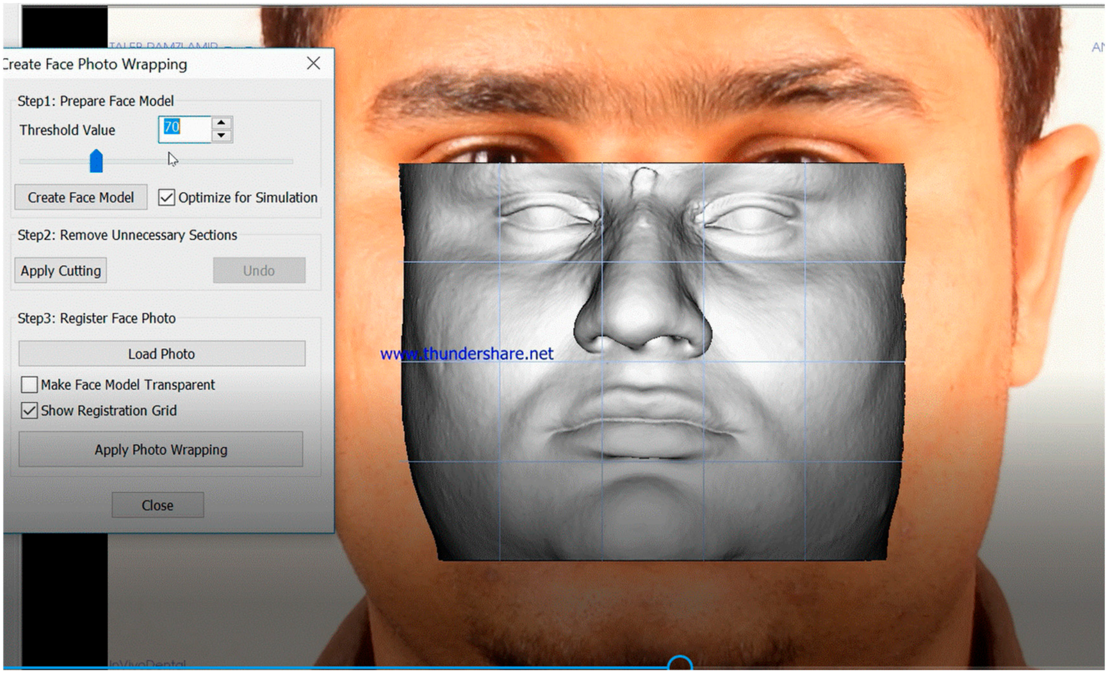

8) **Zoom the 3D volume to be more fit with the loaded photo**

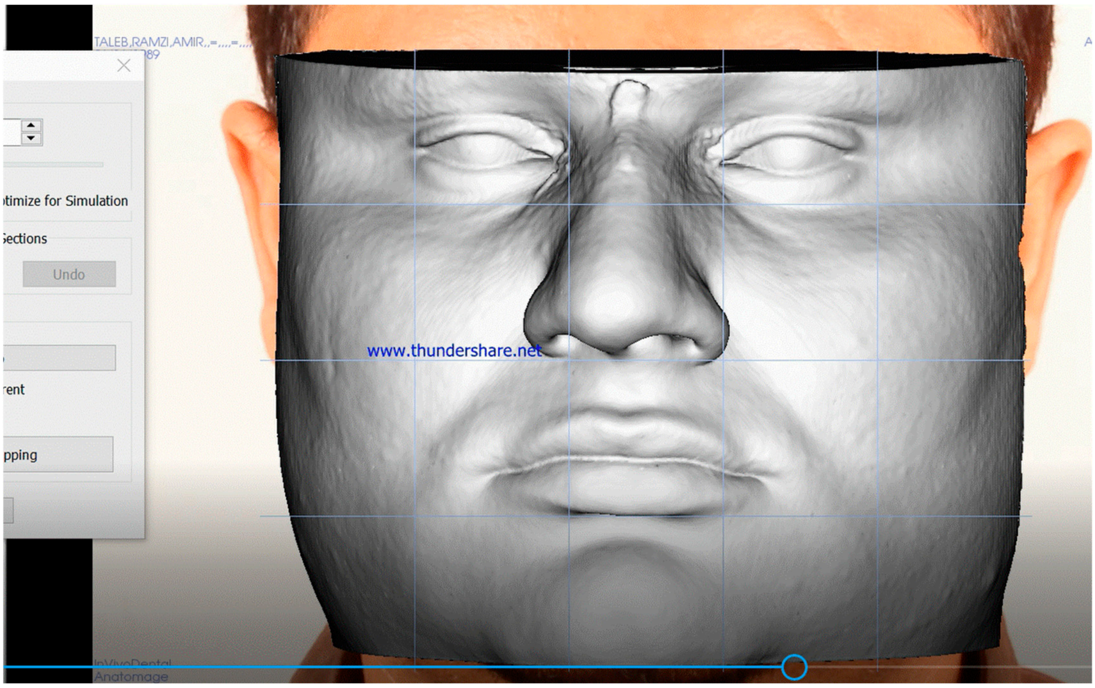

9) **Make face model transparent for easy adjustment and registration of the selected landmarks**

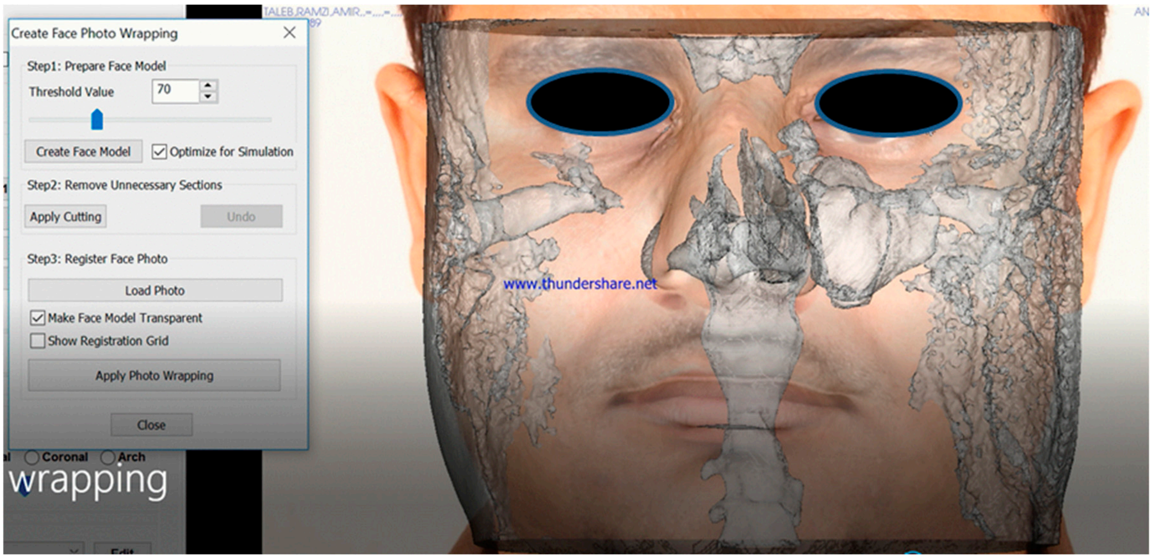

10) **Identify and confirm selected landmarks and do the needful adjustment**

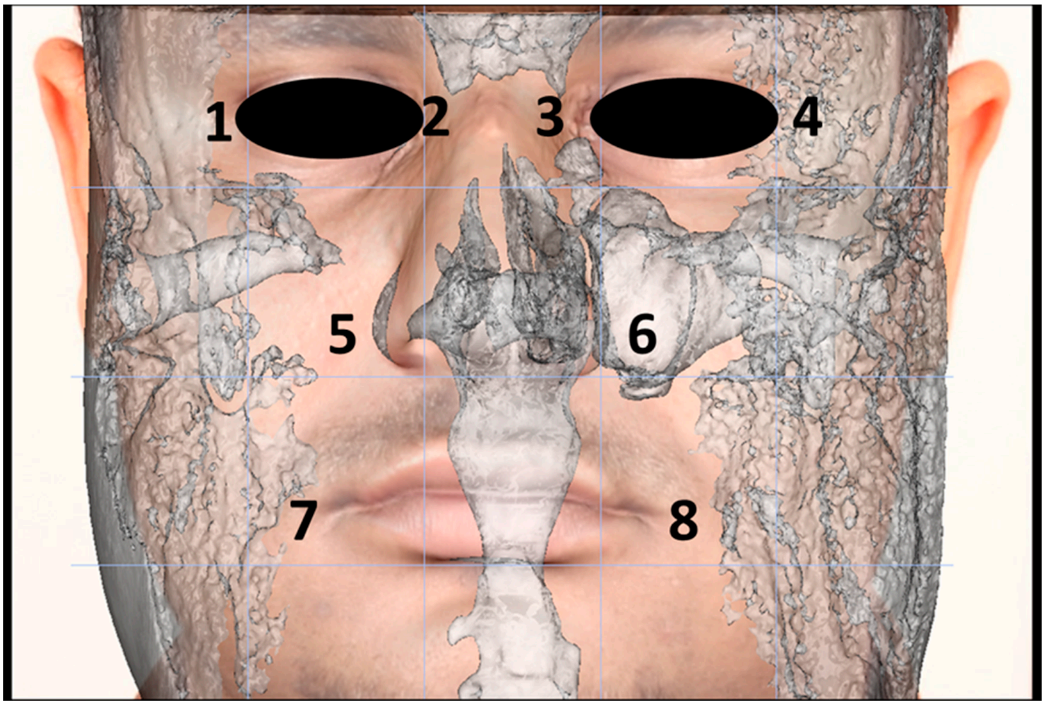

11) Click on apply photo wrapping

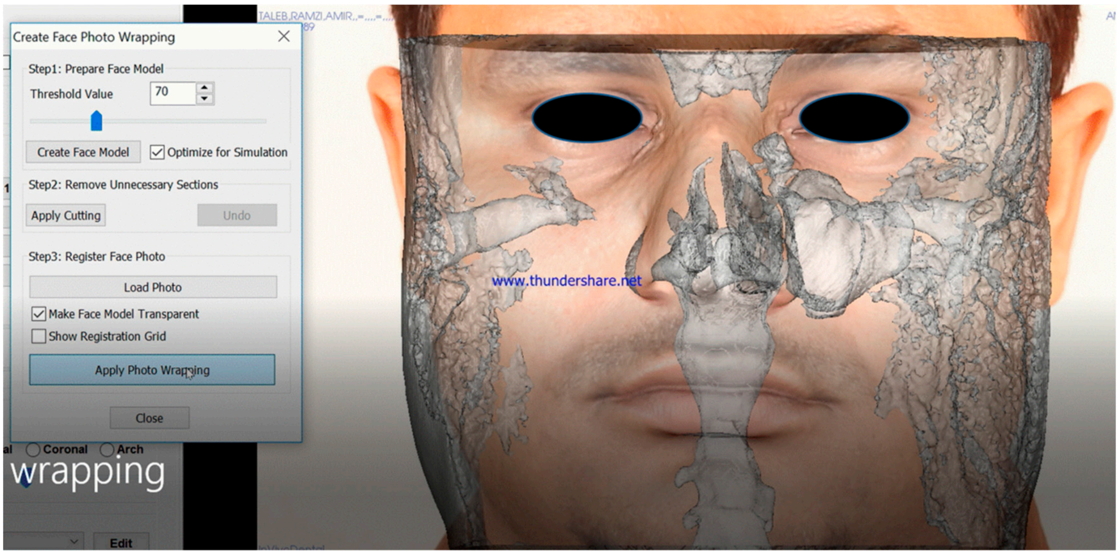

12) Perform 3D analysis on the profile view

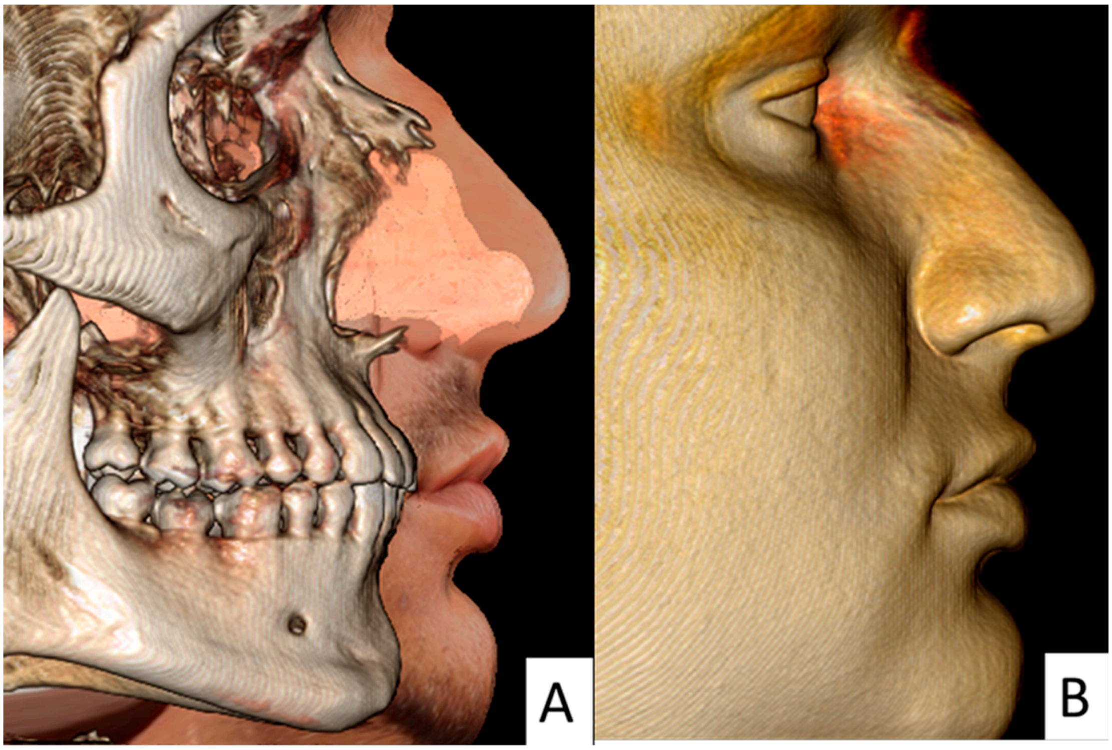

Supplement: Supplementary file 1 [file diagnostics-11-00757-s001.zip › diagnostics-1191623-supplementary.pdf]
